# Laceration of Cervix, Endometritis, Lateral Displacement of Uterus, Pelvic Peritonitis Following Abortion

**Published:** 1893-04

**Authors:** Matthew D. Mann

**Affiliations:** Buffalo, N. Y.; Lecture delivered at the Buffalo General Hospital


### LACERATION OF CERVIX, ENDOMETRITIS, LATERAL DISPLACEMENT OF UTERUS, PELVIC PERITONITIS FOLLOWING ABORTION.

By MATTHEW D. MANN, M. D., Buffalo, N. Y.

Lecture delivered at the Buffalo General Hospital, February 23, 1891, (Reported by A. L. BENEDICT, M. D.)

This patient is aged 35, has been married sixteen years, and has had two children, who are twelve and fourteen respectively. She had no trouble with the labors except that she says they were quite slow, the first being quicker than the second, which is rather the reverse of the rule. On closer questioning, however, she says that there were only about three hours of very hard pains—not a very long time. Six months after the second child was born, she became pregnant, but miscarried at about the second month. She had little pain at the time of the miscarriage, but she was quite sick afterward for four weeks, having fever, and she says she was so low that she could not speak. She was in bed for six weeks, and was two or three months in regaining her strength. This is a common history, for a miscarriage even at the second month may do the greatest amount of harm. She has never been quite well since the miscarriage. Except specific infection, I know of nothing that causes so much pelvic trouble as miscarriages. This woman has been an invalid for twelve years, as the result of that trouble. She complains of backache, headache, pain in the left side and left eye—the pain occurs only occasionally in the right eye, and she cannot see so well with the left eye. She has to wear spectacles to read, and even with them, if she reads for a long time, her eyes trouble her, Her monthly periods cause her a good deal of pain and headache. The headache comes sometimes before, sometimes after, the periods. It is in the back of the head and neck, and it runs down the spine. This is characteristic, and it has been called the uterine headache. It is of the nature of sick headache or hemicrania, although there may be no nausea with it, being really a neuralgia, accompanied by vaso-motor disturbances. Whenever she stands and tries to work, there is a bearing down pain, which she feels especially in the left side. The bowels are costive and she has to take cathartics three or four times every week. The movements of the bowels are painful.

There are some very instructive points in the history of this case. One is the fact that it began with a miscarriage at the second month, and that the patient has never been a well woman since. Evidently at that time she had septicemia from retained bits of membrane, which decomposed in the uterus and set up a septic endometritis. This became, probably, a salpingitis — but, before I go on with these deductions, let me tell you what I find. I could not make an absolute diagnosis in such a case without an examination, and so I examined her before I brought her into the clinic-room. I found that the cervix is very badly lacerated. It is a kind of laceration that would be quite likely to escape notice, for there is a great hypertrophy of the anterior lip, a very slight crease between it and the posterior lip, which is atrophied so that it seems to run into the vaginal wall and it is hardly felt at first. The atrophy is not so marked as would at first appear, it being principally relative, owing to the hypertrophy of the anterior lip. The cervix is sensitive to the touch, and especially in the line of the scar.

The body of the uterus lies well over on the right side and it is very tender. When I squeeze it between my two hands, I can feel it slightly in the median line. The right side of the pelvis is perfectly empty, as far as the uterus is concerned, and it is entirely free from tenderness. This is the reason why I said the woman had a septic endometritis and salpingitis. You never find a uterus drawn down to one side except as the result of septic inflammation. The adhesions which formed in the more acute stage of the inflammation have contracted with age and have drawn the uterus over to the left side and they hold it there. You would not think the woman would suffer so much from the condition, but remember that the rectum runs on this side, and it is very closely connected with this broad ligament and tube. Almost always in the cases of laparatomy in which we have to raise the left ovary and tube, we pull up the sigmoid flexure with them, and almost all cases of salpingitis that have lasted for any length of time are associated with catarrh of the rectum, showing the extension of inflammation from the outside of the peritoneum over the intestine into the mucous membrane.

This woman is suffering from the effects of laceration of the cervix, due to displacement of the uterus, and pelvic peritonitis. It is altogether likely that the associated peritonitis and salpingitis are the worst part of the disease. They are what give her the most suffering, but they are kept up and she is made to suffer more by the laceration, because there is a large eroded surface, which is a constant source of irritation and which keeps up the congestion within the pelvis. Of course, the septic element has all gone, long ago. Probably the quickest way out of the whole trouble would be a laparatomy, but I do not suppose the patient would submit to that and I shall try a simpler method first. I shall propose to sew up the lacerated cervix and see if, by removing this source of irritation, the other trouble will not subside to a certain extent. It is probable that the uterus will become smaller and the old salpingitis and peritonitis will not give her so much trouble as they do now. This restoration of the cervix must be done very carefully. It would not do to drag down the uterus and break those adhesions, for there is danger of setting up a new attack of peritonitis, and the operation must be done with considerable care in order to prevent any possibility of infection. She has been sick so long that she will probably submit to this operation and it will afford her great relief. When there is not the imperative necessity of doing the graver operation first, I always prefer to do the simpler operation first, so as to afford the patient the opportunity of recuperating her strength as much as possible for the more severe ordeal.

Of course, if this woman's tubes are full of pus, it would be nonsense to operate on the cervix.

She is about to have her monthly sickness, and as soon as that is over she promises to come up to the hospital and enter the ward, and I will then operate upon her.

